# Empirical Study of Overfitting in Deep Learning for Predicting Breast Cancer Metastasis

**DOI:** 10.3390/cancers15071969

**Published:** 2023-03-25

**Authors:** Chuhan Xu, Pablo Coen-Pirani, Xia Jiang

**Affiliations:** Department of Biomedical Informatics, University of Pittsburgh, Pittsburgh, PA 15217, USA

**Keywords:** deep learning, overfitting, modeling, breast cancer, metastasis, activate function, weight initializers, network structure, learning rate, momentum beta, iteration-based decay, dropout rate, training epochs, batch size, L1, L2

## Abstract

**Simple Summary:**

It is important to be able to effectively predict the likelihood of breast cancer metastasis to potentially help make treatment plans for a patient. We developed a type of deep learning models called feedforward neural network (FNN) models to predict breast cancer metastasis using clinical data. We found that overfitting can affect the prediction performance negatively, and overfitting and model performance can be greatly affected by hyperparameter settings. In this research, we conducted grid search experiments to study how each of the 11 hyperparameters of our FNN models is related to overfitting and model performance. Our experiment results show that the top five hyperparameters that have a significant impact on overfitting are iteration-based decay, learning rate, batch size, L2, and L1. The experiment results illustrate that different hyperparameters have a different impact on overfitting, and hyperparameter tuning with grid search can help improve the prediction performance of the FNN models.

**Abstract:**

Overfitting may affect the accuracy of predicting future data because of weakened generalization. In this research, we used an electronic health records (EHR) dataset concerning breast cancer metastasis to study the overfitting of deep feedforward neural networks (FNNs) prediction models. We studied how each hyperparameter and some of the interesting pairs of hyperparameters were interacting to influence the model performance and overfitting. The 11 hyperparameters we studied were activate function, weight initializer, number of hidden layers, learning rate, momentum, decay, dropout rate, batch size, epochs, L1, and L2. Our results show that most of the single hyperparameters are either negatively or positively corrected with model prediction performance and overfitting. In particular, we found that overfitting overall tends to negatively correlate with learning rate, decay, batch size, and L2, but tends to positively correlate with momentum, epochs, and L1. According to our results, learning rate, decay, and batch size may have a more significant impact on both overfitting and prediction performance than most of the other hyperparameters, including L1, L2, and dropout rate, which were designed for minimizing overfitting. We also find some interesting interacting pairs of hyperparameters such as learning rate and momentum, learning rate and decay, and batch size and epochs.

## 1. Introduction

Breast cancer is the number one cause of cancer-related deaths for US women aged 20 to 59, estimated to account for 43,600 deaths in 2021 [1,2,3,4,5,6]. Breast cancer metastasis is the main cause of breast cancer death [7]. Being able to effectively predict the likelihood of metastatic occurrence for each individual patient is important because the prediction can guide treatment plans tailored to a specific patient to prevent metastasis and to help avoid under- or over-treatment. 

Deep machine learning methods are now more and more used in healthcare-related prediction tasks including the prediction of breast cancer metastasis [8,9]. A neural network (NN) is one of the machine learning methods that can be used to conduct prediction. An NN consists of layers of artificial neurons, also called nodes, loosely mimicking how the human brain and nervous system work to process and pass signals via neurons [10]. Due to this, an ANN is also referred to as an artificial neural network (ANN). A traditional ANN consists of one input layer, one hidden layer, and one output layer [11]. Deep learning is the use of a non-conventional ANN that is composed of more than one hidden layer, which is also referred to as a deep neural network (DNN) [11].

In this research, we used an EHR dataset concerning breast cancer metastasis to study the overfitting of deep feedforward neural network (FNNs) prediction models. We included 11 hyperparameters of the deep FNN models and took an empirical approach to study how each of these hyperparameters was affecting both the prediction performance and overfitting when given a large range of values. We also studied how some of the interesting pairs of hyperparameters were interacting to influence the model performance and overfitting. We hope, through our study, to help answer some of the questions mentioned in the previous paragraphs.

Our results are consistent with some of the existing research findings or knowledge, such as that activation function is associated with overfitting, the prediction performance tends to drop when a lot of momentum works together with a large learning rate, and a smaller batch size is often associated with better prediction performance. Our results not only substantiate some of the existing knowledge in the field of machine learning but also present interesting new findings. These types of findings are useful for mitigating overfitting when conducting hyperparameter tuning and selecting the range of hyperparameter values required by grid search. We hope to obtain interesting findings that add to our existing knowledge about overfitting and are helpful to the grid search approach of learning, or at least encourage research interests in this direction.

## 2. Method

### Overfitting, Hyperparameters, and Related Work

**Figure 1:**
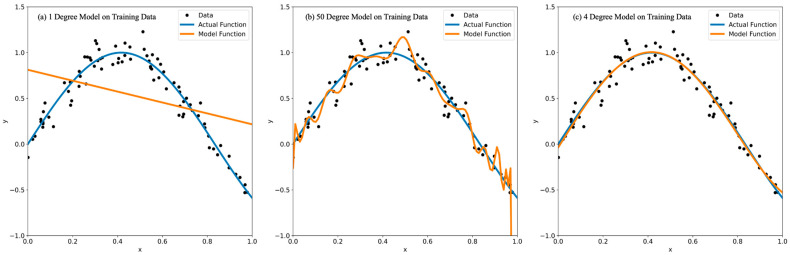
Underfitting and overfitting examples on randomized data with 1, 50, and 4 degree model.

Overfitting occurs when a model performs well on training data but generalizes poorly to unseen data. The reasons for this include the limited size of the training dataset, imbalance in the training dataset, the complexity of models, and so on [12,13]. For example, in Figure 1a,b, there is a generated dataset that has been used to train a polynomial model of varying degrees. Figure 1b is an example of model overfitting because the polynomial function’s degree is too high. Since the function is long and overly complex, it fits to the data too well and begins directly connecting the data points. This is bad because when the model is run on data that it has never seen before, it will predict incorrectly based on the training data and not the testing data. While overfitting is the subject of this paper and arguably more common, its antithesis is also important to consider when training and testing new models [14]. Underfitting is a problem where the model performs badly on both training data and unseen data, generally occurring when a model is overly regularized, inadequately trained, or lacks relevant predictive features [15]. Figure 1a is clearly underfitted as the model has not captured the trend in the data accurately. To fix this, the degree must be raised, but if it is too high then the model is too complex and overfitting occurs, as seen in Figure 1b. By tuning the degree parameter, the model in Figure 1c below can be obtained, which is the optimized function in this case. When looking at an underperforming model, it is important to distinguish between the model being outright incorrect and it being underfitted. For example, if a model is trained for too long and is too complex, the next logical step is to remove features and reduce training time as said model is overfitted. As can be seen from Figure 1a, if these logical steps go too far, it will turn into an underfitted model as a consequence of attempting to prevent overfitting. 

**Figure 2:**
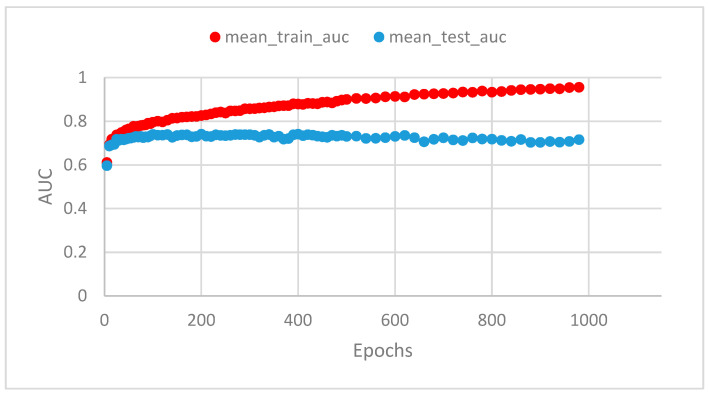
Training epoch with AUCs.

Another example of overfitting and underfitting can be found within this paper, when looking at a graph containing epochs as a hyperparameter, such as Figure 2. “Epochs” define the number of times that the entire training dataset is used by the learning algorithm during training. As seen in Figure 2, the first few epochs will either be emitted or look vastly incorrect, in this case the latter. These sorts of results are a direct case of underfitting as the deep learning model needs multiple full passes of the data to find the pattern and adapt adequately. Additionally, the higher the epochs, the further away from each other the mean_train_AUC and mean_test_AUC become, which means we are encountering overfitting near the end of this graph. This is because the model is performing better and better on training data but worse and worse on testing data. While, the model was underfitted in some cases, in most cases where both the training and test data performance is bad, it will be due to a problem with the model itself. In practice, it is sometimes challenging to strike a balance between training sufficiently and training too much (overfitting). 

It is not possible to completely eliminate overfitting in deep learning, but there are known hyperparameters of deep learning that can be adjusted to reduce the effect of overfitting [16]. As mentioned previously, “epochs”, a hyperparameter that helps balance model convergence, are a known factor of overfitting in deep learning [17]. The “dropout rate” is another hyperparameter that is known to affect overfitting [18]. Neurons are randomly selected and dropped out during training based on the preselected dropout rate to reduce time cost and minimize model overfitting. Additionally, regularization hyperparameters L1 and L2 are known to reduce overfitting [19]. L1, also called a sparsity regularization factor, is a factor that can be used to remove the effect of the “noisy” input nodes and make the network less complex. L2 is a regularization factor based on weight-based decay, which penalizes large weights to adjust the weight updating step during model training. Activation function also plays an important role in preventing the model from overfitting [20]. The role of the activation function in neural networks is to introduce nonlinear factors with nonlinear combinations of weighted inputs to solve problems that cannot be solved by linear models including Sigmoid activation function, ReLu activation function, and so on. Finally, the number of hidden layers is another factor that may impact the overfitting. Every neural network will include multiple layers, with each layer of neurons receiving input from the previous layer and producing output to the next layer, so a large number of hidden layers may lead to an overfitting problem [21]. Deep learning became a popular method due to its success in a lot of image-relevant applications, and part of its success is attributed to its various hyperparameters that can be optimized [22]. 

The process of optimizing the hyperparameters is also called hyperparameter tuning, which often involves selecting the set of hyperparameter values that has the best prediction performance out of all sets of hyperparameter values being tested [23]. The sets of hyperparameter values can either be selected manually or selected automatically following certain rules, and the latter method is often called grid search. Grid search is designed to conduct hyperparameter tuning in a systematic way by going through each of the sets of hyperparameter values automatically during the model training process [23]. Other than epochs, dropout rate, L1, and L2, the four hyperparameters that are known to affect overfitting, various other hyperparameters can be tuned in a grid search of deep learning [24]. The hyperparameter “optimizer” can assume different values such as SGD (stochastic gradient descent) and Adagrad (adaptive gradient descent). SGD adjusts its learning rate via “momentum” and “decay”, the two other hyperparameters that can be tuned via grid search. The momentum is a moving average of the gradients that can help accelerate the convergence of training. The decay is an iteration-based decay factor that can be used to decrease the learning rate in each epoch during the optimization process [25]. The “learning rate” is a hyperparameter that governs how big of a step it takes each time to update the internal model parameters (weights and biases) in response to the estimated error during the model training process [26]. It is used by both the SGD and Adagrad. Adagrad adapts the learning rate to the parameters, conducting smaller-step updates for parameters linked to frequently appearing features and larger-step updates for parameters linked to less frequent features. The batch size is also a hyperparameter of deep learning that controls the number of the training samples that are “fed” into the neural network before internal model parameters are updated [27]. 

In a grid search, each of the hyperparameters is given a preselected series of values, and the program will then iterate through every hyperparameter value combination possible to train models [23]. We call a hyperparameter value combination a hyperparameter setting. There is great uncertainty when choosing a set of values for a hyperparameter when conducting a grid search. For example, the traditional textbook or default value for learning rate is 0.01. However, technically speaking, learning rate can assume numerous values, so should we choose 0.001 to 0.01 with a step size of 0.001 or should we choose 0.0001 to 0.05 with a step size of 0.005? In addition, there is no definite answer as to whether and how much learning rate affects overfitting in deep learning. 

Although some of the hyperparameters such as epochs, dropout rate, L1, and L2 are known to have an influence on overfitting, there are still questions as to which of them has the least or the largest effect. Similar to learning rate, there is still a lot of uncertainty when selecting a range of values for such a hyperparameter, which is required by grid search. For instance, since technically there is no upper bound for the value of number of epochs, you can choose 1 to 500 epochs or 1 to 5000 epochs when conducting grid search. As previously illustrated, when the number of epochs is too low, you can underfit, and when it is too high, you can overfit. Furthermore, when the number of epochs becomes higher, grid search becomes slower, and you can waste a lot of computing time with worse results due to overfitting. So, for these hyperparameters, what are the ranges of values that you can choose to be most likely to avoid underfitting and overfitting? 

Most of the research on deep learning in the medical field is based on image identification, focusing on convolutional neural networks [28]. Empowered with large-scale neural networks and massively parallel computing devices, the accuracy of image recognition is greatly improved [22]. However, image data are only one type of “big data”. There are a large quantity and variety of non-image data that can be very valuable to machine learning and personalized medicine [29]. For instance, the electronic health record (EHR), a widely available data resource, can be utilized for the purpose of tailoring therapies and providing prognostic information. An EHR database contains abundant data about patients’ clinical features, disease status, and clinical outcomes. Such data are invaluable to tailoring diagnoses and prognoses to individuals with diseases such as breast cancer [30,31]. In this research, we used an EHR dataset concerning breast cancer metastasis to study the overfitting of deep feedforward neural network (FNNs) prediction models [31]. We included 11 hyperparameters of the deep FNNs models and took an empirical approach to study how each of these hyperparameters was affecting both the prediction performance and overfitting when given a large range of values. We also studied how some of the interesting pairs of hyperparameters were interacting to influence the model performance and overfitting. We hope, through our study, to help answer some of the questions mentioned in the previous paragraphs. 

## 3. Experiments

In this research, we conducted a unique type of grid search with deep learn repeatedly to study how each of the 11 hyperparameter influences model overfitting and prediction performance when assuming various values. In this type of grid search, we gave a wide range of values to the hyperparameter being studied at the time, while each of the other 10 hyperparameters assumed a single value, which was randomly picked from a set of values. For each of the 11 hyperparameters, we repeated this type of grid search 30 times, so that we could obtain the mean measurements of model overfitting and prediction performance, averaged over 30 values, for each of the range of values given to the hyperparameter of interest. Next, we discuss in detail all aspects of the method we used. The specific steps are as follows:

1. Identify a large range of values for grid search for each of the 11 hyperparameters.
2. Run a unique grid search for each of the 11 hyperparameters, in which we only change the values of the targeted hyperparameter determined in step 1.
3. Repeat the grid search as described in step 2 30 times for each of the 11 hyperparameters.
4. Calculate the mean measurements of model overfitting and prediction performance over 30 values obtained via step 3 for each hyperparameter.

### 3.1. Feedforward Deep Neural Networks (FNNs)

**Figure 3:**
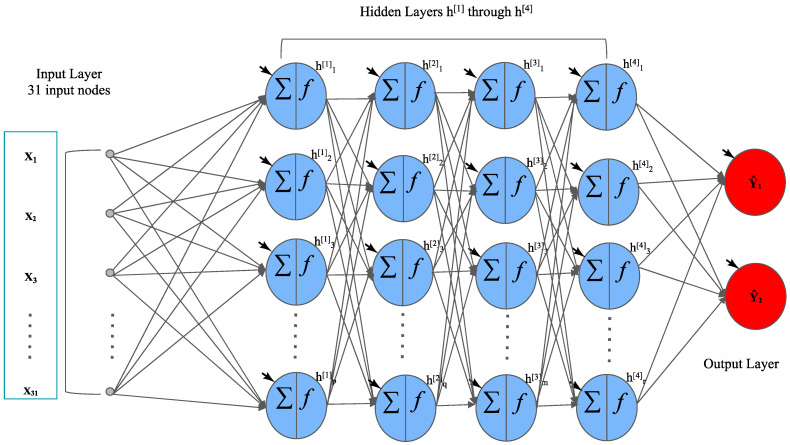
The structure of an FNN model that we developed.

The deep neural networks used in this study are fully connected FNNs that have at least one hidden layer [23]. Inspired by biology, these neural networks do not contain cycles and each data point simply traverses a chain of hidden layers. Figure 3 contains a summary of the inner workings of one of the FNNs that we developed in this study. It includes four hidden layers and two output layers. The 31 input nodes of this neural network represent the 31 clinical features contained in the patient data that will help the model predict breast cancer metastasis, and the output layer contains two nodes representing the binary status of breast cancer metastasis. Each node in the model has an activation function, represented by f(x), which decides the node’s individual output value established by the current value of the node. In both the input layer and the hidden layer(s), we employed a rectifier linear unit (ReLU) as the activation function. In Figure 3, each hidden layer has a certain number of hidden nodes that can be different from the other layers, as can be seen by the distinct variables p, q, m, and r, respectively. The weight matrix of the neural network was initialized using glorot_normal initializer. This FNN model was developed in Python using the TensorFlow and Keras packages. 

### 3.2. A Unique Type of Grid Search Designed for This Study

#### 3.2.1. Single Hyperparameter

We considered 11 hyperparameters including activation function, weight initializers, learning rate, momentum, decay, dropout rate, epochs, batch size, L1, and L2 in this study. For each of the 11 hyperparameters, we conducted a unique type of grid search with deep learning 30 times, and each time, we gave a wide range of values to the one hyperparameter of interest while each of the remaining hyperparameters assumed its base value. The base values of the hyperparameters came from the best model resulting from grid searches in our previous study [23]. Table 1 shows the hyperparameters, their base values, and the values that we tested in this research for the purpose of studying overfitting and underfitting. 

#### 3.2.2. Paired Hyperparameter

We conducted a grid search in which we gave a range of values to a pair of hyperparameters while fixing the values of the remaining hyperparameters, which is sometimes referred to as an interactive grid search in this text. When a lot of momentum combines with a high learning rate, training can change in large steps to pass the global minimum. Therefore, it is believed that a setup where learning rate and momentum have a negative correlation is the best. Due to this, we are interested in knowing how learning rate and momentum interact to affect overfitting and model performance. We are also interested in knowing whether and how learning rate and decay interact to affect overfitting and prediction performance, because they are often used together in the SGD optimizer. We also conducted interactive grid searches with batch size and epochs and with L1 and L2, because batch size and epochs are both related to the number of data points the model “sees” during the training, and L1 and L2 are both regularization methods that adjust the loss function. The fixed values of the remaining hyperparameters are the base values as shown in Table 1. The ranges of values used for two of the hyperparameters in the interactive experiments are shown in Table 2.

### 3.3. Evaluation Metrics and Dataset

#### Measurement for Prediction Performance

AUC is one of the most important metrics for evaluating the classification model performance, and it has been traditionally used in medical diagnosis since the 1970s [32]. The higher the AUC, the better the performance of the model in terms of distinguishing between the positive and negative classes. In normal cases, the valid AUC should be between 0.5 and 1, which means this model will be able to distinguish different classes. 

In this research, the deep neural network was trained on the LSM 5-year dataset that was published in previous studies [30,31]. This dataset contains 4189 patient cases and 31 clinical features that are used as the predictors by the FNN models. The class feature is a binary variable representing whether a patient metastasized within 5 years of the initial treatment. Please refer to Supplementary Table S1 [23] for a detailed description of all the variables included in this dataset. We developed a custom grid search output format that documents 64 output values for each model trained using grid search, including both the results and computer system information, model performance numbers, and total computation time. Each binary diagnostic test outputs a receiver operator characteristic (ROC) curve. The area under the ROC, also known as AUC, has been extensively used in medical diagnosis since the 1970s and is still one of the crucial methods used to judge classification performance in machine learning and deep learning models [32,33]. To compute the AUC of our deep learning model, we used the 5-fold cross validation technique to equally split the dataset into five portions for training and testing. This severance was almost fully random, except we had to ensure that each fraction of the dataset was accurately represented, so around twenty percent of both positive and negative cases were assigned to each portion. We trained and tested the model five separate times, and for each iteration, a different portion acted as the validation dataset to assess the model that was trained on the other four portions. AUC values for both training and testing were recorded for each of the five train/test cycles alongside the average AUC values over all five cycles. We used the best mean test AUC to choose the final hyperparameter values for this study, and the optimized model includes all these best hyperparameter values. The procedure outlined above was used for all experiments and methods conducted in this study. 

### 3.4. Measurement for Overfitting

We used percent_AUC_diff to measure overfitting, a measurement that we introduced in our previous study [23]. It represents the percent difference of the average AUC of the 5 training sets and the average AUC of the 5 testing sets during the 5-fold CV process. The average AUC for training, denoted as mean_train_AUC, and the average AUC for testing, denoted as mean_test_AUC, are both part of the standard output values of a grid search. The specific formula for computing percent_AUC_diff is percent_AUC_diff = (mean_train_AUC − mean_test_AUC)/mean_test_AUC. The mean_train_AUC is expected to be somewhat better than the mean_test_AUC because models are trained by the training sets of data (so called the cyclic effect). However, when the average AUC for the training is significantly higher than the average AUC for testing, or when the percent_AUC_diff is higher than a threshold value such as 5%, we can consider that the model is overfitted.

## 4. Results 

**Figure 4:**
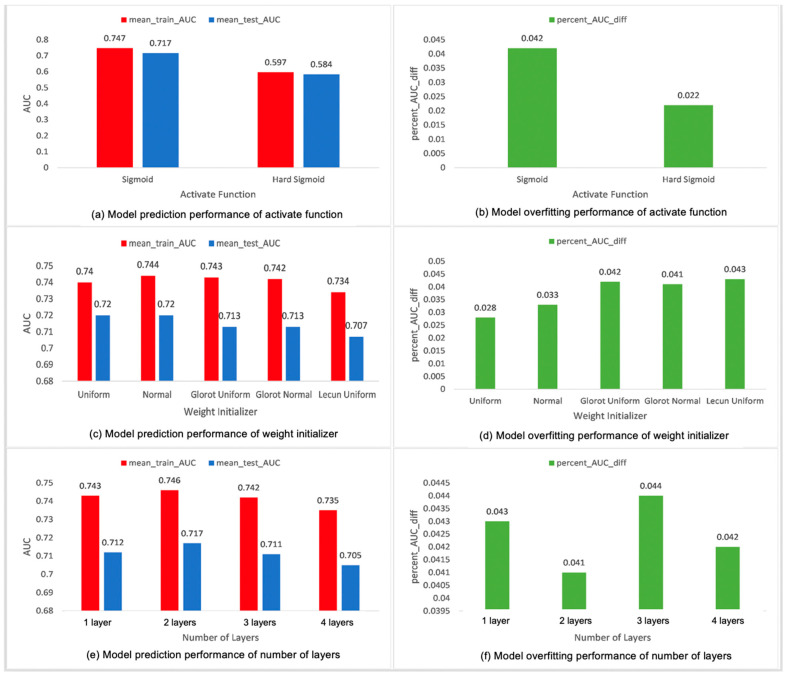
Average mean_train_AUC, mean_test_AUC, and percent_AUC_diff for activation function, weight initializer, and number of layers.

Recall that for each of the hyperparameters, we conducted our unique type of grid search 30 times, and each time we obtained the five-fold cross validation results for each of the models trained during the grid search, including the mean_train_AUC, mean_test_AUC, and percent_AUC_diff. The mean_test_AUC is used to access the prediction performance of a model. Mean_train_AUC is based on the training dataset in a five-fold CV process. Percent_AUC_diff is used to quantify overfitting, and computed based on both mean_train_AUC and mean_test_AUC. The mean_train_AUCs, mean_test_AUCs, and the percent_AUC_diffs shown in our results are the averaged values of the 30 experiments, so they are means of means. Figure 4 is a panel of six figures that shows our averaged grid search results for the hyperparameters activation function, weight initializer, and number of hidden layers. Figure 4a is a bar chart showing the average mean_train_AUCs and mean_test_AUCs, of the normal sigmoid and hard sigmoid, the two values we tested for the activation function of the output layer. Figure 4b shows the matching average percent_AUC_diff for these two values. Hard sigmoid is piecewise linear approximation of the logistic sigmoid activation function, which was designed to reduce the calculation time of the normal sigmoid activation problem [34,35]. Both normal sigmoid and hard sigmoid are suitable for the binary output that we have. Based on Figure 4a,b, the normal sigmoid performs better than hard sigmoid in terms of both mean_train_AUC and mean_test_AUC, but the average percent_AUC_diff of the normal sigmoid is slightly higher than that of the hard sigmoid. The weight initialization method of a neural network has a vital influence on the convergence speed and performance of the model [36]. A good weight initialization can help alleviate the problem of gradient disappearance and gradient explosion [37]. We tested five values: uniform, normal, Glorot_normal, Glorot_uniform, and Lecun_uniform. As shown in Figure 4c, the normal, Glorot_normal, Glorot_uniform, and Lecun_uniform perform better than the two uniform types of weight initializer in terms of the mean_train_AUC and mean_test_AUC. In terms of overfitting, as shown in Figure 4d, the regular uniform and normal weight initializers also perform better than the other three with lower percent_AUC_diff values. We also included the number of hidden layers as one of the hyperparameters, because based on some previous studies, in most cases, the more layers a model has, the more complex the model is and the more likely that the model is doing worse in terms of overfitting [20,38]. Based on our results shown in Figure 4e, the two hidden layers models perform slightly better and the four hidden layers models perform slightly worse than the other models, but overall, all types of models perform similarly. It is worth noting that based on our results in Figure 4f, it is not necessarily true that a model with more layers performs worse in terms of overfitting.

**Figure 5:**
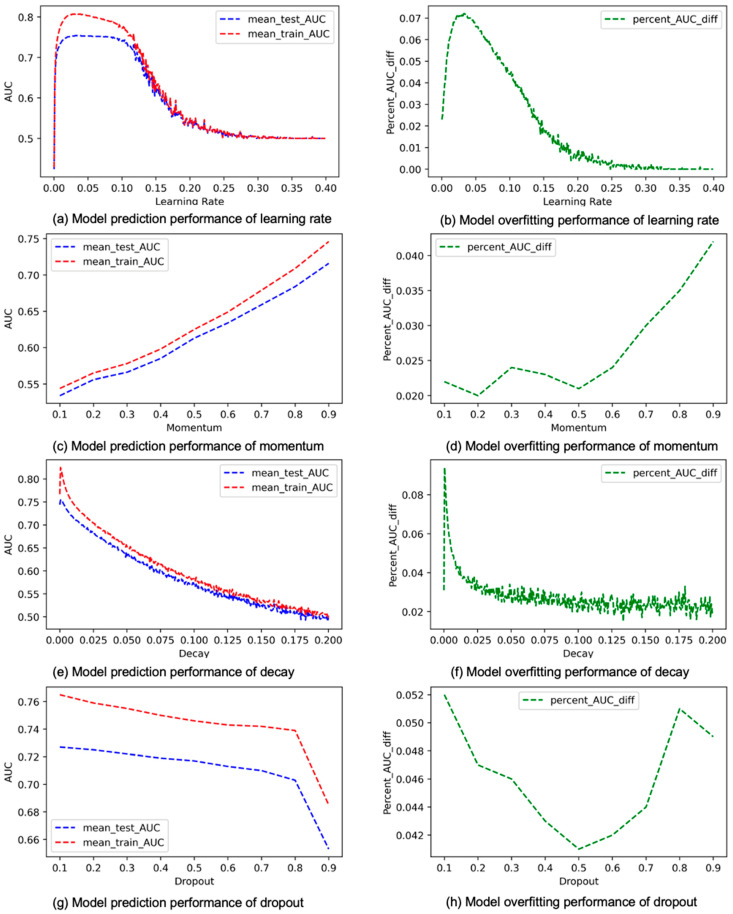
Average mean_train_AUC, mean_test_AUC, and percent_AUC_diff for learning rate, momentum, decay, and dropout.

**Figure 6:**
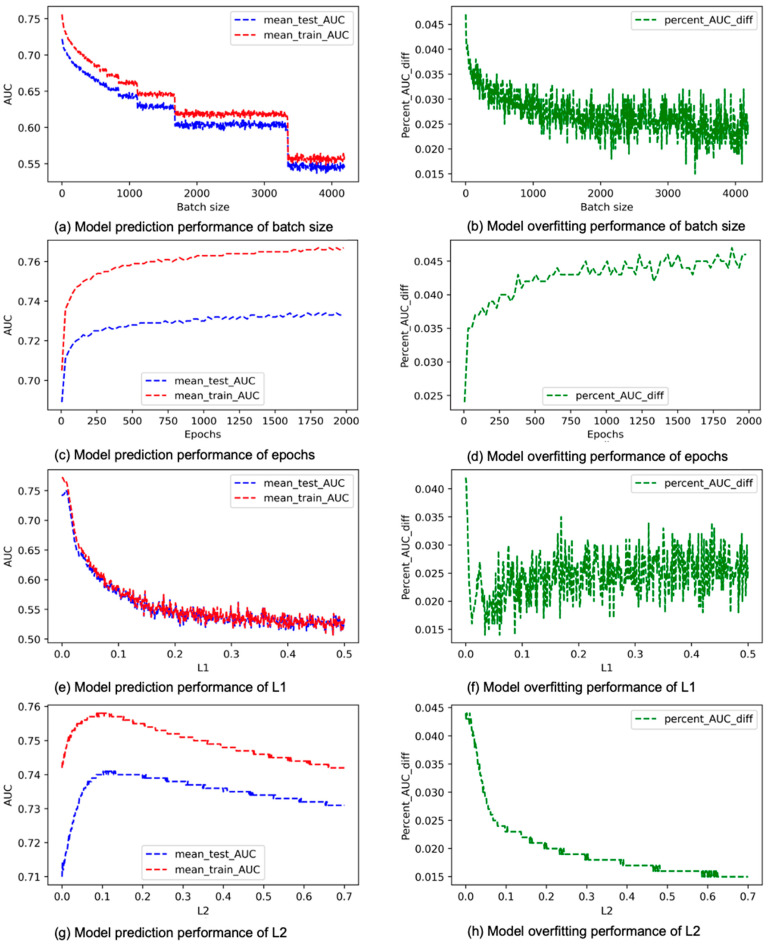
Average mean_train_AUC, mean_test_AUC, and percent_AUC_diff for batch size, epochs, L1, and L2.

The results concerning learning rate, momentum, decay, and dropout appear in Figure 5. Learning rate is used to update weights in the gradient descent procedure during training. According to a study described in [20], the lower the learning rate, the slower the gradient decrease and the easier it is for the model to overfit. Furthermore, learning rate is a critical hyperparameter for striking a balance between elongated convergence time and not converging at all due to gradient explosion [12]. Figure 5a shows the average changes in model performance in terms of mean_test_AUC and mean_train_AUC when the learning rate gradually increases. Figure 5b shows how overfitting changes with learning rate [39]. Based on Figure 5, we tend to obtain the best performing model when the learning rate is between 0.05 and 0.1, because when the learning rate is at this range, the mean_test_AUC remains high while overfitting drops down quickly. According to Figure 5b, when the learning rate is low, the model tends to overfit, and this result is consistent with what was reported in [20]. However, we do notice that overfitting is, in fact, steadily becoming higher before the learning rate reaches 0.05, and then it starts to decrease once the learning rate surpasses 0.05. Momentum is used to add a fraction of preceding weight update to the current weight update to help dampen gradient oscillation when the gradient keeps changing direction. However, when the gradient persistently points to a certain direction for an extended period with a lot of momentum, training can be trapped at a local minimum, which can lead to overfitting. This explains our results in Figure 5d, which demonstrate that model overfitting continuously becomes worse when the momentum increases. In addition, based on Figure 5c,d, when the momentum exceeds 0.5, we tend to obtain a good model that has a high mean_test_AUC without being too overtrained. Decay modifies a model’s loss function in a way that allows it to phase out internal weights that have not been updated recently. This helps reducing the complexity of a model while not affecting performance [40]. Dropout can also help in reducing the complexity of a model and, therefore, reducing overfitting, and it does this by dropping out nodes randomly based on a predetermined probability [41]. Both the performance curves in Figure 5e and the overfitting curve in Figure 5f for decay show a negative correlation. Overall, when decay increases in value, overfitting will decrease, but mostly at the cost of degrading prediction performance. However, we notice that when decay falls in a small range of values, approximately from 0.005 to 0.01, overfitting drops more effectively without damaging the performance as much. This may indicate that we can identify a good model more efficiently when focusing on this small range of values of decay during grid search. The situation for dropout is somewhat more complicated. As shown in Figure 5h, when the dropout rate is below 0.5, it tends to be negatively correlated with overfitting, but when it is greater than 0.5, it becomes mostly positively correlated with overfitting. Figure 5g shows that the prediction performance is negatively correlated with dropout, and this correlation becomes especially strong when the dropout rate reaches 0.8. Figure 6 contains the results for batch size, epochs, L1, and L2 [42]. Batch size is a hyperparameter that determines how many data points the model sees at any given time. The values of batch size we used ranges from 1 to the maximum number of data points contained in the dataset (4186) with a step size of 5. Figure 6a,b shows that both prediction performance and overfitting are negatively correlated with batch size, but overfitting is not significant (less than the 5% threshold) even in the worst case. Based on this result, we tend to obtain the best performing model when the batch size is very small. A possible explanation is that a large batch size feeder tends to converge to sharp minimizers of the training and testing functions and that sharp minimizers lead to poorer generalization. Epochs are the number of times when the entire dataset is seen by the model. When the number of epochs is too low, the model is not trained sufficiently, which causes underfitting. However, as the number of epochs increases, the number of weight updates increases, and the chance of overfitting also increases [43]. According to Figure 6c,d, both prediction performance and overfitting are positively correlated with epochs, while the performance improvement apparently slows down when the number of epochs exceeds 250. The L1 and L2 regularization methods are commonly used in machine learning to control model complexity and reduce overfitting [44,45,46]. Based on Figure 6e,f, we obtain the best-performing model before L1 reaches about 0.03. After that, as L1 increases, the prediction performance degrades quickly, and overfitting tends to become slightly worse instead of better. Figure 6g shows that overfitting is negatively correlated with L2 throughout, and Figure 6h demonstrates that the prediction performance is positively correlated with L2 when L2 is below 0.1, but it become negatively correlated with L2 when L2 exceeds 0.1. Overall, we tend to obtain the best-performing model before L2 reaches 0.1, when prediction performance has not yet been degraded but overfitting is already in the tolerable range. 

**Figure 7:**
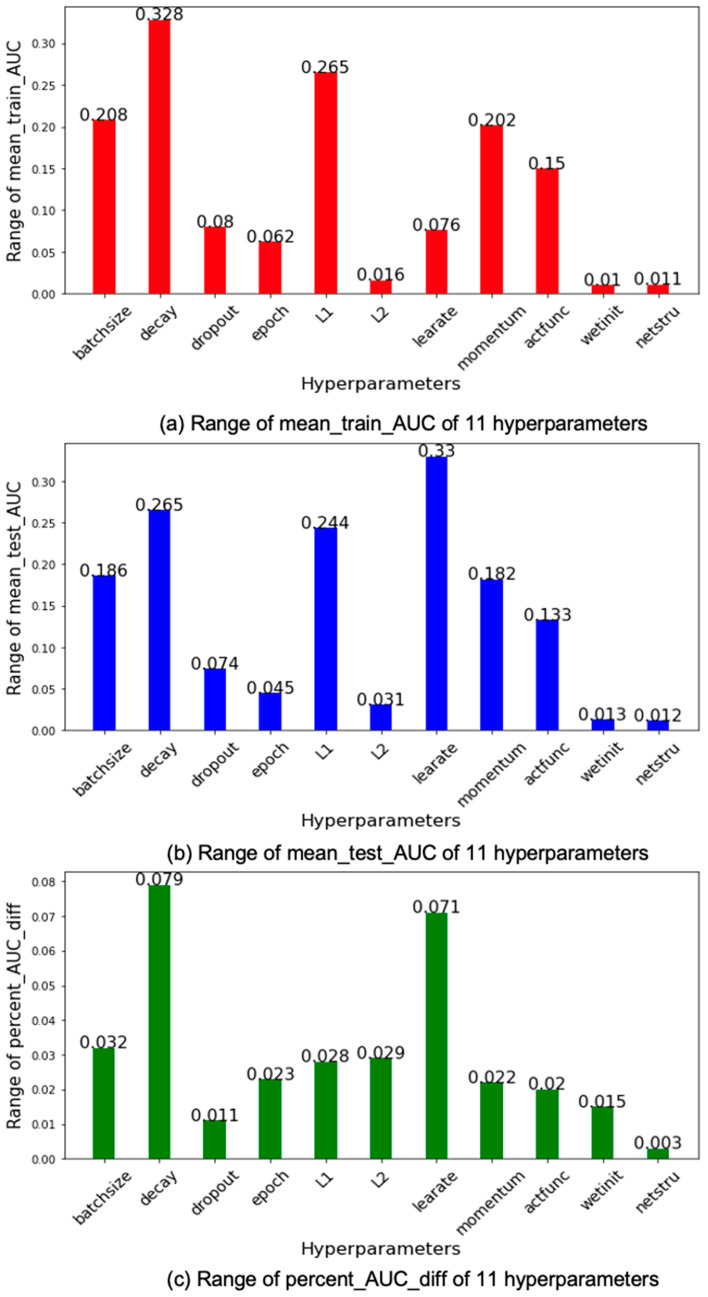
Ranges of the mean_train_AUCs, mean_test_AUCs, and percent_AUC_diff s of the Hyperparameters.

For every grid search we ran for each of the hyperparameters, we obtained the maximum and the minimum values of the mean_train_AUC, mean_test_AUC, and percent_AUC_diff from all the models trained. We then computed the averaged maximums and minimums of the 30 grid searches for each of the hyperparameters. The ranges of mean_train_AUCs, mean_test_AUC, and percent_AUC_diffs for each of the hyperparameters are the differences between the averaged maximums and minimums, which are shown, respectively, in Figure 7a–c. 

According to Figure 7a, the top three hyperparameters in terms of the range of mean_train_AUC are decay (first, 0.328), L1 (second, 0.265), and batch size (third, 0.208). It is not hard to observe that there is a strong correlation between Figure 7a,b, except for hyperparameter learning rate, which has huge range of mean_test_AUC but relatively small range of mean_train_AUC. The bottom three hyperparameters are the same for both mean_test_AUC and mean_train_AUC, including weight initializer, number of hidden layers, and L2. 

Based on Figure 7c, the top three hyperparameters in terms of the range of percent_AUC_diff are decay (first, 0.079), learning rate (second, 0.071), and batch size (third, 0.032). The bottom three are the number of hidden layers (11th, 0.003), dropout rate (10th, 0.011), and weight initializer (9th, 0.015). Based on Figure 7b, the top three hyperparameters in terms of the range of mean_test_AUC are learning rate (first, 0.33), decay (second, 0.265), and L1 (third, 0.244), and the bottom three are number of hidden layers (11th, 0.012), weight initializer (10th, 0.013), and L2 (9th, 0.031).

Based on Figure 7, changing the values of learning rate, decay, and batch size has a more significant impact on both overfitting and prediction performance than doing so with most of the other hyperparameters, including the ones that were designed for the purpose of minimizing overfitting such as L1, L2, and dropout. Overfitting is reported to be associated with a large number of hidden layers [21], but based our results, overfitting is the least sensitive to the number of hidden layers. 

**Figure 8:**
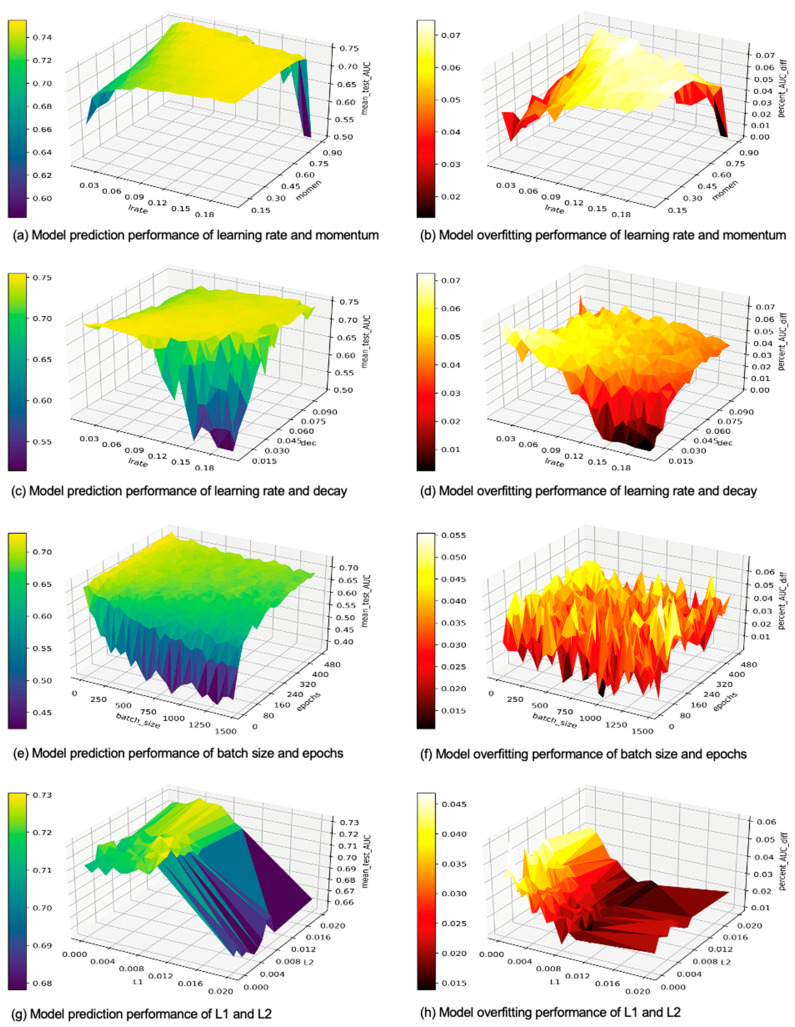
Results of the interactive experiments with hyperparameter pairs.

The results of our paired hyperparameter experiments are shown in Figure 8. It is believed that a setup where learning rate and momentum have a negative correlation is the best [39]. A performance drop is indeed seen in Figure 8a when a lot of momentum combines with large learning rates. Based on Figure 8a,b, we obtained the best mean_test_AUCs but high overfitting when small learning rates combine with a lot of momentum. Figure 8c,d shows that a combination of large learning rates and large values of decay may help improve results, while a large learning rate can result in poor prediction performance when decay is low. Figure 8e,f shows that although prediction performance overall negatively corelates with batch size, it can be increased when a large batch size combines with a large number of epochs. Interestingly, Figure 8g,h seems to indicate that L2 alone does not have a very high impact on either the prediction performance or overfitting, and neither does it seem to interact much with L1.

## 5. Conclusions

Our results not only substantiated some of the existing knowledge in the field of machine learning but also presented interesting new findings. More specifically, our results are consistent with some of the existing research findings or knowledge such as the fact that activation function is associated with overfitting [20], the prediction performance tends to drop when a lot of momentum interacts with a large learning rate [39], and smaller batch size is often associated with better prediction performance. On the other hand, we expected to see that the number of layers is closely associated with overfitting based on the literature [21], but this does not show clearly in our results. In addition, our results show that most of the single hyperparameters are either negatively or positively corrected with model prediction performance and overfitting. In particular, we found that overfitting overall tends to negatively correlate with learning rate, decay, batch size, and L2, but tends to positively correlate with momentum, epochs, and L1. As discussed in the Results section, we also noticed that prediction models are prone to performing better within a certain range of hyperparameter values for some of the hyperparameters. For example, we are more likely to see better results when momentum exceeds 0.5, when drop out is below 0.5, or before L1 reaches 0.02. These types of findings are useful for selecting the range of hyperparameter values required by grid search. According to our results, learning rate, decay, and batch size may have a more significant impact on both overfitting and prediction performance than most of the other hyperparameters, including the ones that were designed for the purpose of minimizing overfitting such as L1, L2, and dropout. 

## Figures and Tables

**Table 1:** Hyperparameters and their values in grid search.

Hyperparameters (Number of Values Tested)	Values Tested	Base Values
1. Activation function for output layer (2)	Sigmoid Hard Sigmoid	Sigmoid
2. Weight initializers (5)	Uniform Normal Glorot Uniform Glorot Normal Lecun Uniform	Glorot_Normal
3. Number of hidden layers (4)	1 layer (75,1) 2 layers (75, 75, 1) 3 layers (75, 75, 75, 1) 4 layers (75, 75, 75, 75, 1)	2
4. Learning rate (400)	0.001–0.4 with step size 0.001	0.005
5. Momentum beta (9)	0.1–0.9 with step size 0.1	0.9
6. Iteration-based decay (400)	0–0.2 with step size 0.0005	0.01
7. Dropout rate (9)	0.1–0.9 with step size 0.1	0.5
8. Training epochs (80)	5–2000 with step size 25	100
9. Batch size (838)	1–4186 (dataset size) with step size 15	10
10. L1 (501)	0–0.5 with step size 0.001	0
11. L2 (701)	0–0.7 with step size 0.001	0.008

**Table 2:** Hyperparameters and their values in interactive grid search.

Paired Hyperparameters	First Hyperparameter (Number of Values)	Second Hyperparameter (Number of Values)
Learning rate and momentum	0.01–0.2 with step size 0.01 (20)	0.1–0.9 with step size 0.005 (9)
Learning rate and iteration decay	0.01–0.2 with step size 0.01 (20)	0.005–0.1 with step size 0.005 (20)
Batch size and epochs	1–1500 at a step size of 50 (30)	5–500 at a step size of 25 (20)
L1 and L2	0–0.01 with step size 0.001 and 0.02 (12)	0–0.01 with step size 0.001 and 0.02 (12)

## Data Availability

The data used in this study are available at datadryad.org (https://doi.org/10.5061/dryad.64964m0).

## Supplementary Materials

The following supporting information can be downloaded at: https://www.mdpi.com/article/10.3390/cancers15071969/s1, Table S1: The variables in the LSDS that are analyzed in the study presented here.
