# Supplementary material for: Empirical Study of Overfitting in Deep Learning for Predicting Breast Cancer Metastasis
Source: Cancers (Basel). 2023 Mar 25;15(7):1969. doi: 10.3390/cancers15071969 (PMC10093528; doi:10.3390/cancers15071969)
Supplement: Supplementary file 1 [file cancers-15-01969-s001.zip › cancers-2270274-supplementary.pdf]

Supplementary Table S1

The variables in the LSDS that are analyzed in the study presented here.

Variables included	Description	Values
race	race of patient	white, black, Asian, American Indian or Alaskan native, native Hawaiian or other pacific islander
ethnicity	ethnicity of patient	not Hispanic, Hispanic
smoking	smoking history of patient	ex smoker, non smoker, cigarettes, chewing tobacco, cigar
alcohol usage	alcohol usage of patient	moderate, no use, use but nos, former user, heavy user
family history	family history of cancer	cancer, no cancer, breast cancer, other cancer, cancer but nos
age_at_diagnosis	age at diagnosis of the disease	0-49, 50-69, >69
menopausal_status	inferred menopausal status	pre, post
side	side of tumor	left, right
TNEG	patient ER, PR, and HER2 negative	yes, no
ER	estrogen receptor expression	neg, pos, low pos
ER_percent	percent of cell stain pos for er receptors	90-100, 0-20, 20-90
PR	progesterone receptor expression	neg, pos, low pos
PR_percent	percent of cell stain pos for pr receptors	90-100, 0-20, 20-90
P53	whether P53 is mutated	neg, pos, low pos
HER2	HER2 expression	neg, pos
t_tnm_stage	prime tumor stage in tnm system	0, 1,2,3,4, IS, 1mic, X
n_tnm_stage	# nearby cancerous lymph nodes	0,1,2,3,4,X
stage	composite of size and # positive nodes	0,1,2,3
lymph_nodes_removed	number of lymph nodes removed	0-11, 12-22, > 22
lymph_nodes_positive	number of positive lymph nodes	0, 1-8 >8
lymph_node_status	patient had any positive lymph nodes	neg,pos
histology	tumor histology	lobular, duct
size	size of tumor in mm	0-32, 32-70, >70
grade	grade of disease	1, 2, 3
invasive	whether tumor is invasive	yes,no
histology2	tumor histology subtypes	IDC, DCIS, ILC, NC
invasive_tumor_location	where invasive tumor is located	mixed duct and lobular, duct, lobular, none
DCIS_level	type of ductal carcinoma in situ	solid, apocrine, cribriform, dcis, comedo, papillary, micropapillary
re_excision	removal of an additional margin of tissue	yes, no
surgical_margins	whether residual tumor	res. tumor, no res. tumor, no primary site surgery
MRIs_60_surgery		yes, no
